# First genome-scale insights into the virulence of the snow mold causal fungus *Microdochium nivale*

**DOI:** 10.1186/s43008-022-00107-0

**Published:** 2023-01-10

**Authors:** Ivan Tsers, Ekaterina Marenina, Azat Meshcherov, Olga Petrova, Olga Gogoleva, Alexander Tkachenko, Natalia Gogoleva, Yuri Gogolev, Evgenii Potapenko, Olga Muraeva, Mira Ponomareva, Viktor Korzun, Vladimir Gorshkov

**Affiliations:** 1 Federal Research Center, Kazan Scientific Center of the Russian Academy of Sciences, Kazan, Russia 420111; 2 Laboratory of Computer Technologies, ITMO University, Saint Petersburg, Russia 197101; 3 Institute of Evolution, University of Haifa, 3498838 Haifa, Israel; 4 Department of Evolutionary and Environmental Biology, University of Haifa, 3498838 Haifa, Israel; 5 Bioinformatics Institute, Saint Petersburg, Russia 197342; 6 KWS SAAT SE & Co. KGaA, 37555 Einbeck, Germany

**Keywords:** Phytopathogenic fungi, Hybrid genome assembly, Snow mold, *Microdochium nivale*, Virulence factors, Transcriptome profiling, Extracellular enzymes

## Abstract

Pink snow mold, caused by a phytopathogenic and psychrotolerant fungus, *Microdochium nivale,* is a severe disease of winter cereals and grasses that predominantly occurs under snow cover or shortly after its melt. Snow mold has significantly progressed during the past decade, often reaching epiphytotic levels in northern countries and resulting in dramatic yield losses. In addition, *M. nivale* gradually adapts to a warmer climate, spreading to less snowy territories and causing different types of plant diseases throughout the growing period. Despite its great economic importance, *M. nivale* is poorly investigated; its genome has not been sequenced and its crucial virulence determinants have not been identified or even predicted. In our study, we applied a hybrid assembly based on Oxford Nanopore and Illumina reads to obtain the first genome sequence of *M. nivale*. 11,973 genes (including 11,789 protein-encoding genes) have been revealed in the genome assembly. To better understand the genetic potential of *M. nivale* and to obtain a convenient reference for transcriptomic studies on this species, the identified genes were annotated and split into hierarchical three-level functional categories. A file with functionally classified *M. nivale* genes is presented in our study for general use. *M. nivale* gene products that best meet the criteria for virulence factors have been identified. The genetic potential to synthesize human-dangerous mycotoxins (fumonisin, ochratoxin B, aflatoxin, and gliotoxin) has been revealed for *M. nivale*. The transcriptome analysis combined with the assays for extracellular enzymatic activities (conventional virulence factors of many phytopathogens) was carried out to assess the effect of host plant (rye) metabolites on the *M. nivale* phenotype. In addition to disclosing plant-metabolite-upregulated *M. nivale* functional gene groups (including those related to host plant protein destruction and amino acid metabolism, xenobiotic detoxication (including phytoalexins benzoxazinoids), cellulose destruction (cellulose monooxygenases), iron transport, etc.), the performed analysis pointed to a crucial role of host plant lipid destruction and fungal lipid metabolism modulation in plant-*M. nivale* interactions.

**Supplementary Information:**

The online version contains supplementary material available at 10.1186/s43008-022-00107-0.

## INTRODUCTION

Snow mold (SM) is a severe disease of winter cereals and grasses, developed mostly under snow cover and caused by fungi and fungi-like organisms, which combine both phytopathogenic and psychrotolerant/psychrophilic properties (Matsumoto and Hsiang 2016). This disease is distributed predominantly in the Northern Hemisphere: in Europe, Canada, the United States, the United Kingdom, and Japan (Hsiang et al. 1999; Bankina et al. 2012). In the Central European part of Russia, SM often exceeds the epiphytotic level and, in some years, may cause almost complete loss of winter crops (Sheshegova 2015; Utkina et al. 2019; Ponomareva et al. 2019; 2021; Shchekleina 2020). In Canada, the prevention of SM accounts for around 50% of the yearly fungicide use on turfgrass (Hsiang et al. 1999). SM is one of the most difficult-to-manage plant diseases due to the absence of SM-resistant plant cultivars, the very few donors of quantitative SM-resistance used in breeding programs, challenges with fungicide application because of the development of SM under the snow cover, and poor knowledge of the SM-causative agents (Ponomareva et al. 2021).

**Fig. 1:**
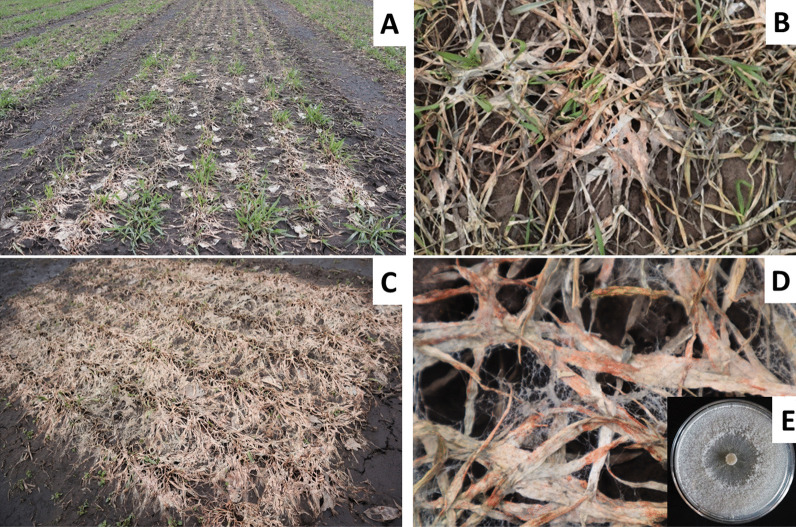
Pink snow mold caused by *Microdochium nivale* on winter rye. Photos were taken on 26.04.2021, ten days after snow melt, in the fields of the Tatar Scientific Research Institute of Agriculture (a subdivision of the Federal Research Center “Kazan Scientific Center of the Russian Academy of Sciences”), located in the center of the European part of Russia in the Laishev District of the Tatarstan Republic, in Bolshiye Kaban (latitude 55.649 N, longitude 49.3083 E, 60 m above sea level). **A**, **B**—moderate disease level, **C**, **D**—high disease level, **E**—*M. nivale* strain F_00608 used in our study

The dominant SM causal fungi belong to the species of two genera of Ascomycota—*Microdochium* (*M. nivale*, *M. majus*), causing pink SM (Fig. 1), and *Sclerotinia* (*S. borealis*), causing snow scald, and one genus of Basidiomycota—*Typhula* (*T. ishikariensis*, *T. incarnata*), causing gray and speckled SM, respectively (Matsumoto and Hsiang 2016). *T. ishikariensis*, *T. incarnata*, and *S. borealis* are psychrophilic organisms that cause diseases only at low temperatures (usually under snow cover). These three psychrophilic species are less distributed and less devastating than the psychrotolerant species *M. nivale* able to cause diseases not only at low (0 to + 4 °C) but also at moderate (+ 15 to + 20 °C) temperatures (Hoshino et al. 2009).

SM has been long considered to be restricted to areas with prolonged snowy winters (Russia, Belarus, Canada, Nordic and Baltic countries) (Parry et al. 1995; Smiley 1996; Johansson et al. 2003). However, pink SM-causing *M. nivale* continuously adapts to warmer weather and shorter winters, allowing SM progression in less snowy territories of Western and Eastern Europe where long periods of cool (+ 4 to + 15 °C) and rainy weather compensate for the absence of snow cover (Matsumoto 2009; Dubas et al. 2011; Zhukovskiy et al. 2020; Ponomareva et al. 2021). Additionally, *M. nivale* is able to cause not only SM but also foot rot, leaf blight, head blight, and other types of diseases throughout the growing period (Tronsmo et al. 2001; Gagkaeva et al. 2017; 2019).

Despite their great economic importance, SM causal fungi, including *M. nivale*, are poorly investigated. *M. nivale* (Fr.) Samuels and Hallett (Samuels and Hallett 1983) was first described in 1825 as *Lanosa nivalis* species (Noble and Montgomerie 1956). Then, due to its similarity with *Fusarium* species, this fungus was attributed to *Fusarium nivale* Ces. ex Berlese and Voglino (Wollenweber 1931). High genetic and phenotypic heterogeneity of different strains, including their differential virulence and host specificity, is typical of *M. nivale* species (Mahuku et al. 1998; Abdelhalim et al. 2020; Gorshkov et al. 2020). Therefore, *M. nivale* is a valuable model for studying not only phytopathogenicity but also the intraspecific diversity and population heterogeneity of fungi, as well as the genetic markers of their phenotypic traits. However, these studies are hampered by the lack of a sequenced and annotated reference *M. nivale* genome. To date, sequences of only a few *M. nivale* conservative loci conventionally used for taxonomic/phylogenetic classification of fungi (18/28S rRNA, internal transcribed spacers (ITS), β-tubulin, elongation factor 1-α, and RNA polymerase subunits) are available in public databases, as well as of several other genes (encoding glyceraldehyde 3-phosphate dehydrogenase (GAPDH), alternative oxidase, aminoadipate reductase, and cytochrome B). No virulence-related genes of *M. nivale* have been sequenced, and the set of these genes in this species remains unknown.

The genomes of many phytopathogenic fungi have been sequenced, resulting in breakthroughs in their study and control. For example, whole-genome sequencing revealed effector protein-encoding genes in *Ustilago maydis* (Kämper et al. 2006), *Botritys* species (Valero-Jiménez et al. 2019), *Zymoseptoria tritici* (Badet et al. 2020), and *Rhizoctonia solani* (Kaushik et al. 2022), as well as mycotoxin-biosynthetic pathways in *Aspergillus fumigatus* and *Elsinoë arachidis* (Gardiner and Howlett 2005; Jiao et al. 2021). Whole-genome analysis enabled the elaboration of valuable diagnostic markers for detecting *Magnaporthe oryzae* and *Calonectria* species (Pieck et al. 2017; Malapi-Wight et al. 2016) and allowed the identification of markers of the differential aggressiveness of *Fusarium graminearum* strains that deserve to be considered for improving diagnostic approaches (Alouane et al. 2021). Reference genomes also form the basis for transcriptomic studies directed toward an understanding of the physiological responses of fungi at a whole genome level (O'Connell et al. 2012; Zumaquero et al. 2019; Chittem et al. 2020; Tang et al. 2021).

Therefore, our study aimed to obtain and annotate the reference *M. nivale* genome sequence, predict virulence factors of this fungus, and assess the influence of the host plant metabolites on the genome expression and the activities of potential virulence factors.

## MATERIALS AND METHODS

### Fungal strain and culture conditions

A highly virulent strain, *Microdochium nivale* F_00608 from the collection of the Laboratory of Plant Infectious Diseases of the Kazan Institute of Biochemistry and Biophysics – Subdivision of the Federal Research Center «Kazan Scientific Center of Russian Academy of Sciences», was used in this study (NCBI BioSample accession SAMN26062287). The strain was isolated from the SM-damaged winter rye grown in field of the Tatar Scientific Research Institute of Agriculture—Subdivision of the Federal Research Center «Kazan Scientific Center of Russian Academy of Sciences», located in the forest-steppe area of the Volga region, Laishev District, Tatarstan Republic, Russia (latitude 55.649 N, longitude 49.3083 E, 60 m above sea level) (Gorshkov et al. 2020). *M. nivale* was cultured submerged in the liquid synthetic medium (LSM) that contained: sucrose (20 g/L), KH_2_PO_4_ (0.7 g/L), K_2_HPO_4_ (0.7 g/L), MgSO_4_ × 7H_2_O (0.7 g/L), NH_4_NO_3_ (1 g/L). NaCl (5 mg/L), FeSO_4_ × 7H_2_O (2 mg/L), KI (0.83 mg/L), H_3_BO_3_ (6.3 mg/L), MnSO_4_ × 4H_2_O (23.3 mg/L), ZnSO_4_ × 7H_2_0 (0.86 mg/L), Na_2_MoO_4_ × 2H_2_O (0.25 mg/L), CoCl_2_ × 6H_2_O (0.25 mg/L), CuSO_4_ × 5H_2_O (0.25 mg/L), CaCl_2_ (0.44 mg/L), mesoinositol (100 mg/L), thiamine (1 mg/L), and pyridoxine (2 mg/L), pH 6.4. The cultivation was performed at 20 °C in darkness without shaking.

To assess the effect of the host plant (rye) extract on the transcriptomic profile of *M. nivale* and its extracellular enzymatic activities, the fungal strain was cultured in LSM for 10 days. Then, 1/10 (v/v) of distilled water or 1/10 (v/v) of water extract of rye leaves was added to the fungal cultures. Cultural supernatants were collected for measuring enzymatic activities, and fungal mycelium was collected for RNA isolation in five biological replicates prior to and 12, 24, 48, and 96 h after the addition of rye extract (or water) to fungal cultures. To obtain the rye extract, 100 g of fresh rye leaves (grown in vermiculite under a 16/8 light/dark period for 14 days) were thoroughly ground in three volumes (w/v) of distilled water, and the obtained suspension was filtered through gauze. The remaining debris was ground in two volumes of water and filtered. Two portions of the obtained filtrates were combined and centrifuged (10,000 g, room temperature, 10 min). The supernatants were collected, incubated for 10 min at 80 °C, and centrifuged again. Then the extracts were sterilized through nitrocellulose filters with pores of 0.22 μm in diameter (Corning Inc., Corning, NY, USA) under sterile conditions and kept frozen (**− **20 °C) until use.

### DNA and RNA library preparation and sequencing

DNA was extracted from *M. nivale* mycelium grown submerged in LSM using the phenol–chloroform method (Sambrook et al. 2012). DNA samples were treated with 100 µg/mL RNase A (Qiagen, Hilden, Germany) and 50 µg/mL proteinase K (Qiagen, Germany) (Maghini et al. 2021). Polysaccharide impurities were removed from the DNA samples with cetyltrimethylammonium bromide (Sigma-Aldrich (Merck KGaA) Burlington, MA, USA) (Góes-Neto et al. 2005). The quantity and quality of the DNA samples were assessed using an Implen nanophotometer (Implen, GmbH, München, Germany) and 1% agarose gel-electrophoresis, respectively. For Illumina sequencing, the DNA was fragmented (the average fragment length was 450 bp) using an M220 focused-ultrasonicator (Covaris, LLC, Woburn, MA, USA). DNA libraries were prepared using the NEBNext Ultra II kit (New England Biolabs, Ipswich, MA, USA). The efficiency of DNA fragmentation and the quality of DNA libraries were estimated using an Agilent 2100 Bioanalyzer and a High Sensitivity DNA kit (Agilent Technologies, Inc., Santa Clara, CA, USA). The sequencing was performed on an Illumina HiSeq 2500 platform (Illumina, Inc., San Diego, CA, USA) at the Joint KFU–Riken Laboratory, Kazan Federal University (Kazan, Russia).

For Oxford Nanopore sequencing, DNA molecules shorter than 10,000 bp were eliminated using the Short Read Eliminator Kit (Circulomics, Inc., Baltimore, MD, USA). DNA libraries were prepared using the Ligation 1D ONT SQK-LSK109 sequencing kit (Oxford Nanopore Technologies, Ltd, UK). The libraries were sequenced by MinION Mk1 (Oxford Nanopore Technologies, Ltd, UK).

For RNA-Seq analysis, total RNA was extracted from *M. nivale* mycelium grown for 10 days submerged in LSM and then sustained in the presence or absence of rye extract for 24 h (for the comparative transcriptome analysis and for the identification of protein-encoding *M. nivale* genes), as well as from axenically grown rye plants (cultivar Ogonek) infected with *M. nivale* 20 days post infection (Gorshkov et al. 2020) (for the identification of the protein-encoding *M. nivale* genes). Fungal mycelium or plant material was ground in liquid nitrogen. Total RNA was extracted using the ExtractRNA Reagent (Evrogen, Moscow, Russia) according to the manufacturer’s instructions. Residual DNA was eliminated using a DNA-free kit (Life Technologies, Carlsbad, CA, USA). RNA quantity and quality (RNA Quality Number, RQN > 7) were analyzed using an Implen nanophotometer (Implen, Germany) and a Qsep100 DNA Analyzer (Bioptic, New Taipei City, Taiwan), respectively. For RNA-Seq, total RNA (1 µg) was enriched with mRNA using the NEBNext Poly(A) mRNA Magnetic Isolation Module (New England Biolabs, USA). Then, mRNA was processed using the NEBNext Ultra II Directional RNA Library Prep Kit for Illumina (New England Biolabs, USA) according to the manufacturer’s instructions. The quality and quantity of the cDNA libraries before sequencing were monitored using an Agilent 2100 Bioanalyzer (Agilent Technologies, USA) and a CFX96 Touch Real-Time PCR Detection System (Bio-Rad, Hercules, CA, USA), respectively. For the quantification of the cDNA libraries, the EVA Green I PCR-Kit (Synthol, Moscow, Russia) and primers for Illumina adapters (Evrogen, Moscow, Russia) were used; PhiX Control (Illumina, USA) was used as a concentration standard. Libraries were sequenced in three biological replicates. Sequencing was conducted on an Illumina HiSeq 2500 (Illumina, USA) at the Joint KFU–Riken Laboratory, Kazan Federal University (Kazan, Russia).

### Hybrid genome assembly, analysis and annotation

The obtained Nanopore reads were filtered for minimum length (1000 bp) and quality (q = 8) using Nanofilt (De Coster et al. 2018). The Illumina raw reads were quality-checked using FastQC (https://www.bioinformatics.babraham.ac.uk/projects/fastqc/) and MultiQC (Ewels et al. 2016). Illumina reads were filtered using fastp (Chen et al. 2018) with the default settings.

**Fig. 2:**
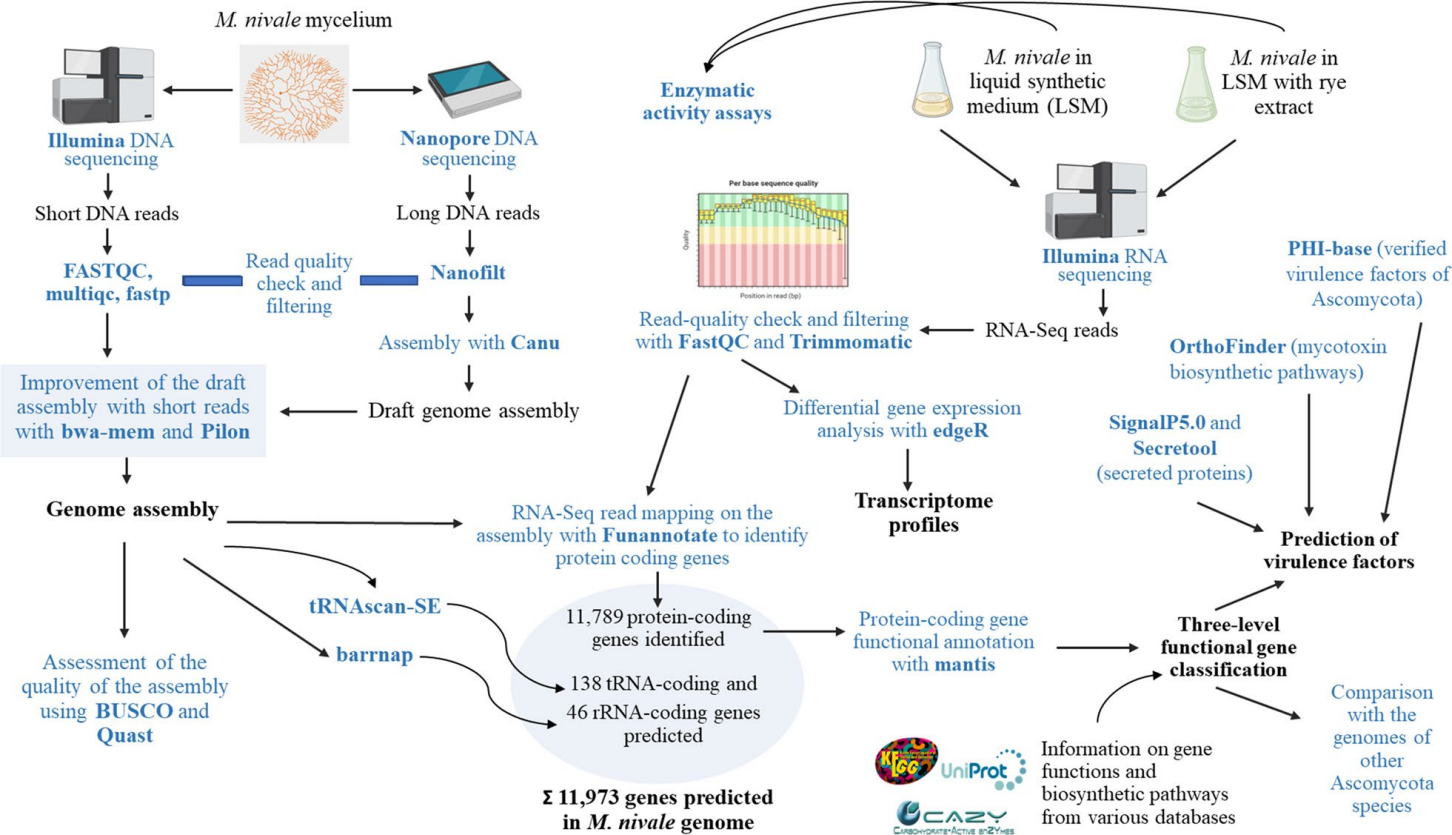
The workflow of the present study

The genome of *M. nivale* was assembled with the Canu assembler v.1.8 (Koren et al. 2017). The Illumina short reads and Nanopore long reads were combined using bwa-mem (Li 2013) and a “polish assembly” by Pilon (Walker et al. 2014). Contiguity and completeness of assembly were assessed with QUAST (Gurevich et al. 2013) and BUSCO (Seppey et al. 2019), respectively. The boundaries of the mRNA transcripts were located via Illumina RNA-Seq read mapping with Funannotate (https://github.com/nextgenusfs/funannotate). The tRNA- and rRNA-encoding genes were predicted using the tRNAscan-SE (Chan and Lowe 2019) and barrnap (https://github.com/tseemann/barrnap), respectively (Fig. 2). Genomic features corresponding to redundant transcripts (duplicates and perfect fragments) were removed using EvidentialGene software with the default settings (Gilbert 2019).

The functions of the *M. nivale* proteins were predicted with the Mantis program (Queirós et al. 2021) using sets of Hidden Markov Models of reference proteins of Ascomycota from PFAM, TCDB, NCBI, and KEGG databases. For each *M. nivale* query, only one reference hit with the highest score (and the lowest e-value) was kept for the annotation; all duplicated reference hits were deleted. The information from the GO, KEGG, KOG, and CAZy databases was combined with the annotation of the *M. nivale* genes and used to perform the automatic classification of genes into functional categories using the R programming language. The merged classification based on the above-mentioned databases was created, manually checked, and edited (Fig. 2).

### The prediction of *M. nivale* virulence factors and a search for the orthologous proteins in other species of Ascomycota

To predict *M. nivale* virulence factors, we used BLAST to align its translated CDS sequences against a set of selected reference proteins from the Pathogen Host Interactions database (PHI-base, http://www.phi-base.org) (Urban et al. 2020). The reference set contained 1009 gene products that were found to affect the virulence of 31 Ascomycota species pathogenic to monocot plants. The best BLAST hits (alignment query coverage ≥ 70%, sequence similarity ≥ 60%) were combined with the gene classification and data on gene expression.

SignalP5.0 (with a likelihood threshold of 0.95) and the Secretool pipeline (with standard settings) were used to predict secreted proteins of *M. nivale* (Cortázar et al. 2014; Almagro Armenteros et al. 2019). Proteins with signaling sequences predicted by both programs were considered secreted proteins.

OrthoFinder with default settings (Emms and Kelly 2019) was used to search for *M. nivale* proteins orthologous to the reference proteins related to major mycotoxin biosynthesis pathways. Biosynthetic pathways were analyzed for the following mycotoxins: (1) trichothecene toxins of *Fusarium sporotrichioides*, including calonectrin (BioCyc pathway ID: PWY-7711), deoxynivalenol (BioCyc pathway ID: PWY-7712), nivalenol (BioCyc pathway ID: PWY-7713), and T-2 toxin (BioCyc pathway ID: PWY-7730); (2) versicolorin B (BioCyc pathway ID:PWY-5955) and aflatoxins of *Aspergillus parasiticus* (BioCyc pathway ID: PWY-5961); (3) gliotoxin of *Aspergillus fumigatus* (BioCyc pathway ID: PWY-7533); (4) fumonisins of *Fusarium verticillioides* (according to the description of the UniProt entry Q8J2Q6); (5) ochratoxins of *Aspergillus niger* (BioCyc pathway ID: PWY-8116); (6) zearalenone of *Fusarium graminearum* (according to the description of the UniProt entry A0A098D6U0); (7) patulin of *Penicillium expansum* (BioCyc pathway ID: PWY-7490). Sequences of all reference proteins related to the listed pathways were retrieved from the SwissProt database (Fig. 2).

Orthologous groups of proteins were identified using OrthoFinder with default settings. The orthologs were searched in the reference proteomes of *M. nivale* and three other Ascomycota species, *M. bolleyi*, *Sclerotinia borealis*, and *Fusarium graminearum* with contrasting lifestyles: *M. bolleyi* is a non-psychrotolernat and non-phytopathogenic species (Rothen et al. 2018), *S. borealis* is a snow mold-causing psychrophilic species (Hoshino et al. 2010), and *F. graminearum* is a mesophilic head blight causal agent (Goswami and Kistler 2004).

### Differential gene expression analysis

The quality of the RNA-Seq reads was assessed using FASTQC (Andrews 2010). Reads of low quality (q < 30) and rRNA-corresponding reads were filtered out using Trimmomatic and SortMeRNA, respectively (Kopylova et al. 2012). Pseudo-alignment and quantification of filtered reads were carried out using Kallisto (Bray et al. 2016) with default parameters and reference transcript sequences of *M. nivale* identified in this study. The edgeR package (Robinson et al. 2010) was used to reveal differentially expressed genes (DEGs). Genes that had TMM-normalized read counts per million (CPM) values ≥ 1 in all replicates within at least one of the experimental conditions were considered to be expressed in our study. Genes with |log_2_FC|> 1 and FDR < 0.05 were considered to be DEGs. The RNA-Seq data were analyzed and interpreted with the application of a functional classification of *M. nivale* genes performed as described above (Fig. 2).

### Enzymatic activity assays

Extracellular enzymatic activities were measured in the supernatants of *M. nivale* cultures prior to and 12, 24, 48, and 96 h after the addition of rye extract (or water). The amylase, cellulase (endoglucanase), xylanase, invertase, and polygalacturonase activities were determined by measuring the reducing sugars released after enzymatic hydrolysis of the corresponding substrates: soluble starch (Sigma-Aldrich, USA) for amylase activity (Sakthi et al. 2012), carboxymethyl cellulose (Sigma-Aldrich, USA) for cellulase activity (Paula et al. 2019), the beechwood xylan (Sigma, USA) for xylanase activity (Nagar et al. 2012), sucrose (Sigma-Aldrich, USA) for invertase activity (Cairns et al. 1995), and polygalacturonic acid (Sigma-Aldrich, USA) for polygalacturonase activity (Kühnel et al. 2011). DNS reagent (Sigma-Aldrich, USA) was used to measure the reducing sugars at 540 nm (Miller 1959). One unit (U) of activity was defined as the amount of enzyme releasing 1 µmol of reducing sugars/min per mg of protein.

Protease activity was determined at 440 nm using azocasein (Sigma-Aldrich, USA) as a substrate. One unit of protease activity was defined as the amount of enzyme required to produce an absorbance change of 1.0 per min per 1 mg of protein (Akpinar and Penner 2002). Lipase and β-galactosidase activities were measured at 410 nm using *p*-nitrophenyl butyrate and *p*-nitrophenyl β-galactoside (Sigma-Aldrich, USA) as substrates, respectively. The amount of enzyme required to produce 1 pmol of *p*-nitrophenol per minute per 1 mg of protein was defined as a unit of lipase or β-galactosidase activity (Margesin et al. 2002; Hofgaard et al. 2006). Pectate lyase activity was determined by measuring the degradation of polygalacturonic acid (Sigma-Aldrich, USA) into unsaturated products at 234 nm (Shevchik et al. 1997). One unit of pectate lyase activity was defined as the amount of enzyme releasing 1 µmol of unsaturated products/min per mg of protein. The absorbance in all enzymatic assays was measured using PB2201B spectrophotometer (SOLAR, Minsk, Belarus). All activities were assayed in five biological replicates.

## RESULTS

### Genome sequencing and assembly

**Fig. 3:**
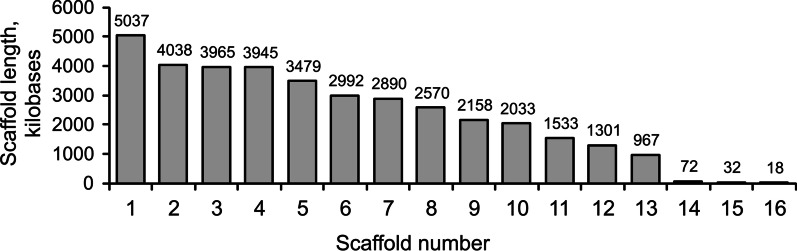
The lengths of scaffolds in the *Microdochium nivale* genome assembly

The details on the Nanopore and Illumina sequencing are given in the Additional file 2: Table S1. The hybrid assembly based on Oxford Nanopore and Illumina reads yielded a *M. nivale* genome with a total length of 37,029,846 bp that were distributed among 16 scaffolds, 12 of which were longer than 1 Mb (Fig. 3). The assembly has been deposited at GenBank under the accession JANTFD000000000, a part of the NCBI BioProject PRJNA808256. The version described in this paper is version JANTFD010000000. The assembly can be alternatively accessed at https://doi.org/10.17605/OSF.IO/2TKUF. Nanopore and Illumina reads covered the genome ~ 55-fold and ~ 76-fold, respectively. The five largest scaffolds contained half of the assembly bases (L50 = 5). According to the results of BUSCO analysis (Benchmarking Universal Single-Copy Orthologs, Seppey et al. 2019), the completeness of the assembled genome was 97.6%: 3,727 complete Sordariomycetes’ BUSCOs were found in the genome, while 90 BUSCOs were fragmented or missed.

### Annotation and functional classification of *M. nivale* genes

To identify the *M. nivale* protein-encoding genes, reads obtained from Illumina mRNA-Seq experiments were mapped onto the assembled genome using the Funannotate pipeline. In total, 11,789 protein-encoding genes were identified. In addition, 138 tRNA- and 46 rRNA-encoding genes were predicted in silico based on a Hidden Markov Model search. The mean length of a *M. nivale* gene was 2,033 bp. Thus, the L50 scaffold (N50 = 3,479,159 bp) was nearly 1,711 times larger than the mean gene size, pointing to the long-range continuity of the assembly.

Functional annotations were assigned to 8,886 M*. nivale* protein-encoding genes; the other 2,903 genes had non-informative annotations (hypothetical proteins and domain of unknown function (DUF)-containing proteins). Based on gene ontologies, metabolic pathway mappings from KEGG, and annotations from UniProt and other databases, the annotated *M. nivale* genes were sorted into 13 functional supercategories, which were further split into categories and then subcategories (Table 1, Additional file 3: Table S2). For example, the supercategory “Plant cell wall modification” was split into 6 categories related to the modification of different plant cell wall components (cellulose, cross-linking glycans, pectin, cutin, and others). In turn, the ‘Cross-linking glycan modification’ category was branched into ‘Arabinan degradation’, ‘Expansins’, ‘Mannan degradation’, ‘Xylan degradation’, and ‘Xyloglucan degradation’ subcategories. The three-level functional classification of all *M. nivale* genes is presented in Additional file 3: Table S2, which can be widely used to improve and simplify the description and interpretation of transcriptomic data on *M. nivale*.

**Table 1:** Summary of the functional classification of *M. nivale* genes

Supercategory	Number of categories	Number of subcategories	Number of attributed genes
Primary metabolism	13	84	3021
Transport	11	27	919
Stress-related	10	13	439
Secondary metabolism	8	17	427
Cytoskeleton	5	5	213
Signaling	9	12	196
Reproduction	3	3	141
Plant cell wall modification	5	11	140
Fungal cell wall (FCW)	4	9	124
Other	–	–	2718
Unknown or hypothetical proteins	–	–	2903
Transcription factors	–	–	581
Cell cycle-related	–	–	161

### Prediction of genes encoding *M. nivale* virulence factors

Initially, to predict genes encoding *M. nivale* virulence factors, we used information from the Pathogen Host Interactions database (PHI-base, http://www.phi-base.org) (Urban et al. 2020), which contains a list of proteins encoded by genes whose impairment reduced the virulence of different pathogens. In PHI-base, 1,009 of such proteins were revealed for those Ascomycota species (31 species) that cause diseases in monocot plants. For 361 of these, similar proteins (BLAST coverage ≥ 70%, sequence similarity ≥ 60%) were found to be encoded by *M. nivale* genes (Additional file 4: Table S3). Most of them were related to the functional categories ‘Primary metabolism’, ‘Cell transport’, and ‘Signaling’ (Additional file 4:Table S3). Here, plant cell wall degrading enzymes (PCWDEs), typical virulence factors of phytopathogens, appeared to be underrepresented: only two genes for *M. nivale* PCWDEs (MN608_08996 and MN608_09345, encoding xylanases) had similarities with PHI-base-listed virulence factors of Ascomycota phytopathogens of monocots.

Then, since many known fungal virulence factors are secreted proteins, we predicted the secretome of *M. nivale*. In total, 869 and 491 secreted proteins were predicted by SignalP5.0 and Secretool, respectively. Both programs predicted 400 M*. nivale* proteins as secreted (Additional file 5: Table S4). Among these 400 proteins (assigned as secreted), 44 PCWDEs (cellulase-like and cutinase-like proteins, as well as a range of cross-linking-glycan- or pectin-degrading enzymes), other carbohydrate-metabolism-related proteins (fructan β–fructosidase, β–glucosidases, β–galactosidase, hexoaminidase-like protein, and two aldose 1-epimerases), 23 proteases, 11 tyrosinases, 7 lipase-like proteins, and 4 hydrophobins were present.

*M. nivale* is considered a species that does not synthesize mycotoxins (Tronsmo et al. 2001; Chełkowski et al. 1991; Nakajima and Naito 1995; Gagkaeva et al. 2020)—microfungi-produced low-molecular-weight non-volatile secondary metabolites that cause disease or death in humans and animals (Bennett and Klich 2003; Dai et al. 2021) and may serve as virulence factors and/or antibiotics (Hof and Kupfahl 2009; Pfliegler et al. 2020). However, the annotation of the *M. nivale* genome revealed many genes related to toxin and antibiotic synthesis, e.g., 17 genes for polyketide synthases (enzymes involved in the biosynthesis of polyketide mycotoxins), 24 aflatoxin biosynthetic genes, and 28 genes annotated as aspirochlorine biosynthesis cytochrome P450 monooxygenase (Additional file 3: Table S2).

**Fig. 4:**
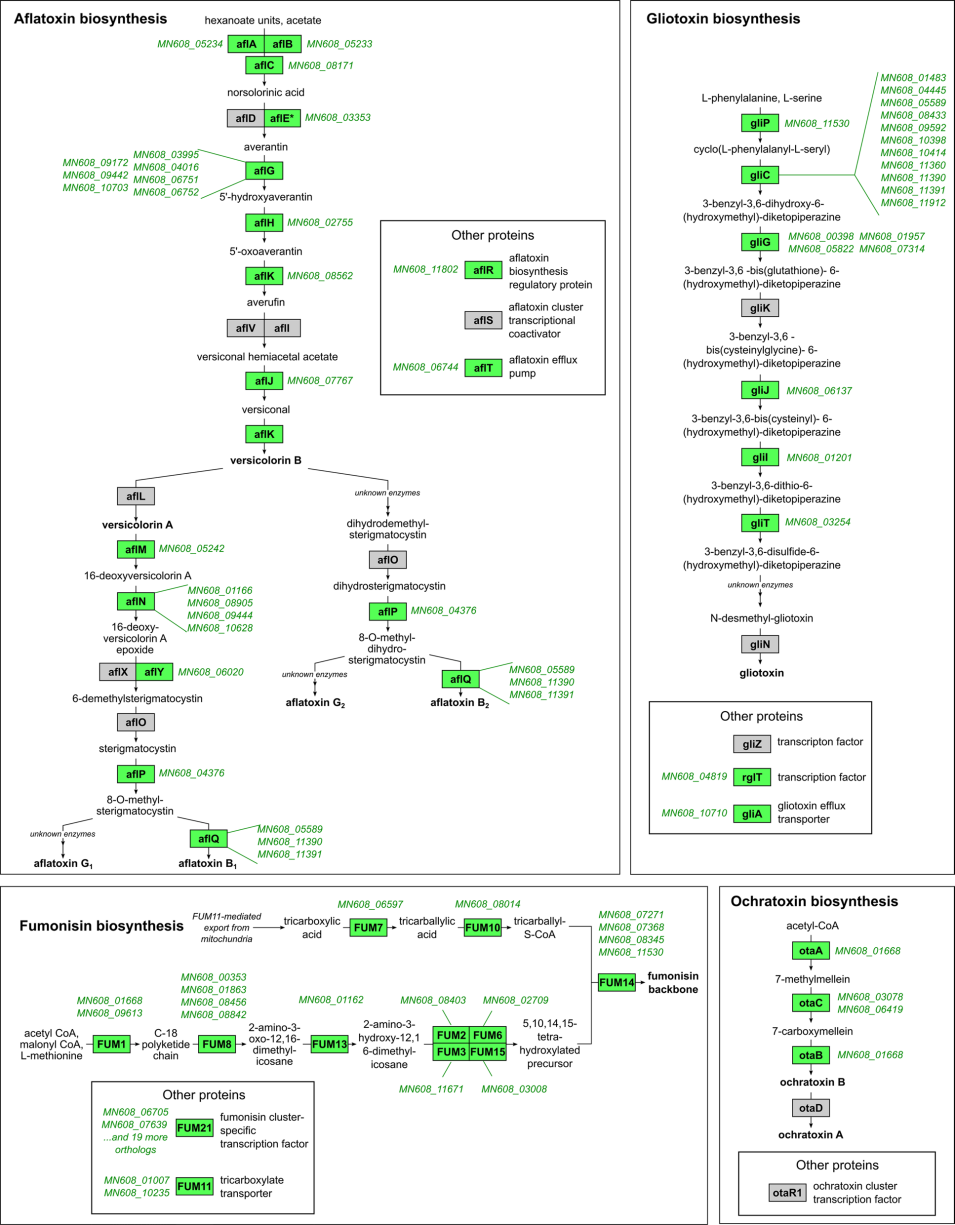
Mycotoxin biosynthetic pathways (yielding fumonisin, gliotoxin, aflatoxins, ochratoxins) predicted in this study to occur in *Microdochium nivale*. Rectangles contain names of the reference proteins involved in the synthesis of corresponding mycotoxins, including those for which orthologs were revealed (green) or were not revealed (grey) in the *M. nivale* proteome. The IDs of genes for the revealed orthologs are given near the corresponding rectangles

To investigate the potential of *M. nivale* for mycotoxin biosynthesis, we searched for the orthologs of the reference proteins (enzymes, transcription factors, transporters) related to the biosynthesis of several major mycotoxins (trichothecene toxins, aflatoxins, gliotoxin, ochratoxin, patulin, and zearalenone (Bennett and Klich 2003) in the *M. nivale* reference proteome. Orthologs for all proteins related to fumonisin and ochratoxin B (but not ochratoxin A) biosynthesis and almost all proteins related to aflatoxin and gliotoxin biosynthesis were revealed in *M. nivale* (Fig. 4, Additional file 6: Table S5). In turn, only some proteins involved in the synthesis of the trichothecene toxins (including dioxynivalenol (DON) and T-2 toxin), patulin, and zearalenone were encoded in the *M. nivale* genome (Additional file 6: Table S5, Additional file 1: Figure S1). Thus, *M. nivale* has an apparent genetic potential to produce mycotoxins such as fumonisin, aflatoxin, ochratoxin B, and gliotoxin (or closely related mycotoxins). The pathways for DON, patulin, zearalenone, and ochratoxin A biosynthesis were partial (incomplete) in *M. nivale*, and therefore, whether these “defective” pathways yield toxic compounds or not remains to be determined.

A list of genes encoding potential *M. nivale* virulence factors (predicted using PHI-base (Additional file 4: Table S3), secreted proteins (Additional file 5: Table S4), and toxin-biosynthetic enzymes (Additional file 6: Table S5)) is presented in the Additional files 4, 5, 6: Tables S3-S5 combined with the functional classification described above (Table 1, Additional file 3: Table S2). The list includes 886 proteins, which deserve special attention in terms of assessing their roles in *M. nivale* virulence.

### Comparison of the reference proteome of *M. nivale* with those of other species

**Fig. 5:**
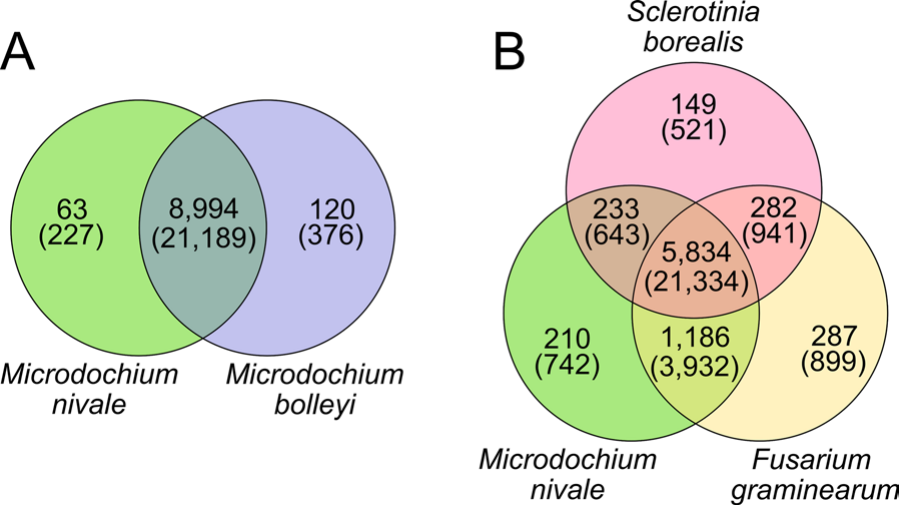
Groups of orthologous proteins (orthogroups) unique to *Microdochium nivale* or shared between **A** *M. nivale* and *M. bolleyi* or **B** *M. nivale*, *Sclerotinia borealis*, and *Fusarium graminearum*. The numbers of the shared orthogroups are given within the corresponding areas, and the numbers of proteins within them are given in parentheses

To assess similarities and differences in genome-encoded protein spectra between *M. nivale* and closely related species as well as other cereal phytopathogens (that cause disease under low or moderate temperatures), the reference proteome of *M. nivale* was compared with the reference proteomes of *M. bolleyi* (the only species of the *Microdochium* genus with a sequenced genome (David et al. 2016)), *Sclerotinia borealis* (the only phytopathogen among psychrophilic/phychrotolerant fungi with a sequenced genome (Mardanov et al. 2014)), and *Fusarium graminearum* (one of the most characterized mesophilic phytopathogens of cereals (Goswami and Kistler 2004)). The comparison of protein sequences of *M. nivale* with those of *M. bolleyi*, a plant root-colonizing non-phytopathogenic and non-psychrotolerant species, revealed 8,994 groups of shared orthologs, containing 21,189 proteins (Fig. 5A), 10,356 of which belong to *M. nivale*. Almost half of the total shared orthologs (10,286) belonged to functional categories related to basal cellular processes (‘Primary metabolism’, ‘Cell cycle regulation’, ‘Fungal cell wall synthesis’, ‘Cellular signaling’, ‘Cytoskeleton organization’). *M. nivale* and *M. bolleyi* also shared a wide range of proteins that are potential virulence factors. Orthologs of *M. nivale* expansin, 43 secreted PCWDEs, secreted lipases and proteases, phytohormon-related proteins (salicylate hydroxylases, gibberellin dioxygenases, an auxin efflux carrier and an auxin hydroxylase), polyketide synthases, toxin-biosynthetic enzymes (aflatoxin, gliotoxin, and fumiquinazolines), and antibiotic-biosynthetic enzymes (aspirochlorine and pentalenolactone) were found in *M. bolleyi* (Additional file 7: Table S6, sheet “Shared”).

In turn, 63 orthogroups of 227 proteins and 120 orthogroups of 376 proteins were unique for *M. nivale* and *M. bolleyi* (compared with each other), respectively (Fig. 5A, Additional file 7: Table S6, sheets “Unique to *M. bolleyi*” and “Unique to *M. nivale*”). Many proteins that were unique to *M. nivale* (compared to *M. bolleyi*) belonged to two functional groups: secondary metabolism (45 unique proteins) and heterokaryon incompatibility (HET) domain-containing proteins, which prevent the development of heterokaryotic mycelium if it is formed by the fusion of hyphae of vegetatively incompatible strains (34 unique proteins). Within the category ‘Secondary metabolism’, 5 ent-kaurene oxidases, 6 hydroxyversicolorone monooxygenases (related to aflatoxin biosynthesis), and 24 aspirochlorine biosynthesis-related cytochrome P450 monooxygenases were revealed. The vast majority of *M. bolleyi'*s unique (compared to *M. nivale*) proteins were unannotated hypothetical proteins. The two largest unique orthogroups of annotated proteins in *M. bolleyi* were ‘P-loop containing nucleoside triphosphate hydrolase proteins’ and ‘amino acid permeases’.

The comparison of the reference proteome of *M. nivale* with those of *S. borealis* and *F. graminearum* revealed 5,834 groups (of 21,334 proteins) of orthologs shared by the three species (Fig. 5B). The largest portion of the shared proteins was functionally related to the ‘Primary metabolism’ supercategory (7,152). The proteins of the functional supercategories ‘Transport’ (1,998), ‘Transcription factors’ (1,060), ‘Stress’ (804), ‘Secondary metabolism’ (593), and ‘Cell cycle’ (404) also appeared to be highly conservative in the three species (Additional file 8: Table S7, sheet “Shared”). The three species also shared many potential virulence factors, including 24 secreted PCWDEs (in particular, cellulose monooxygenase-like proteins, cellobiases, xylanases, pectate lyases), 4 secreted lipases, 8 secreted peptidases, proteins putatively related to phytohormone modification (salicylate hydroxylases, gibberellin dioxygenases, an auxin efflux carrier and an auxin hydroxylase), polyketide synthases, as well as toxin- (aflatoxin, gliotoxin, and fumiquinazolines) and antibiotic-biosynthetic enzymes (gliotoxin/aspirochlorine biosynthesis thioredoxin reductase and pentalenolactone synthase). Herewith, 742 proteins of *M. nivale* had no orthologs in *S. borealis* or *F. graminearum*. Among these were 37 HET-domain containing proteins and 28 oxygenases putatively related to the biosynthesis of aspirochlorine (aspirochlorine biosynthesis cytochrome P450 monooxygenase) (Additional file 8: Table S7, sheet “Unique to *M. nivale*”).

### Host plant extract-induced alterations in the *M. nivale* transcriptome and extracellular enzymatic activities

To assess the effect of the host plant metabolites on *M. nivale* genome expression, the transcriptomic profile of *M. nivale* cultures sustained in the presence of the rye leaf extract was compared with that of cultures sustained without the rye extract. Such a test system allowed the assessment of the effect of plant metabolites on both *M. nivale* gene expression and its extracellular enzymatic activities (the well-known virulence factors of phytopathogens) and thus enabled the comparison of the results of transcriptomic and biochemical analyses.

First, to determine the period at which the effect of rye extract was the greatest, we assayed the dynamics of the extracellular enzymatic activities (cellulase, xylanase, polygalacturonase, pectate lyase, β-galactosidase, amylase, invertase, protease, and lipase). The activities were measured at 12, 24, 48, and 96 h after the addition of rye extract or water (control) to the *M. nivale* cultures. No assayed enzymatic activities were detected in sterile rye extract (data not shown).

**Fig. 6:**
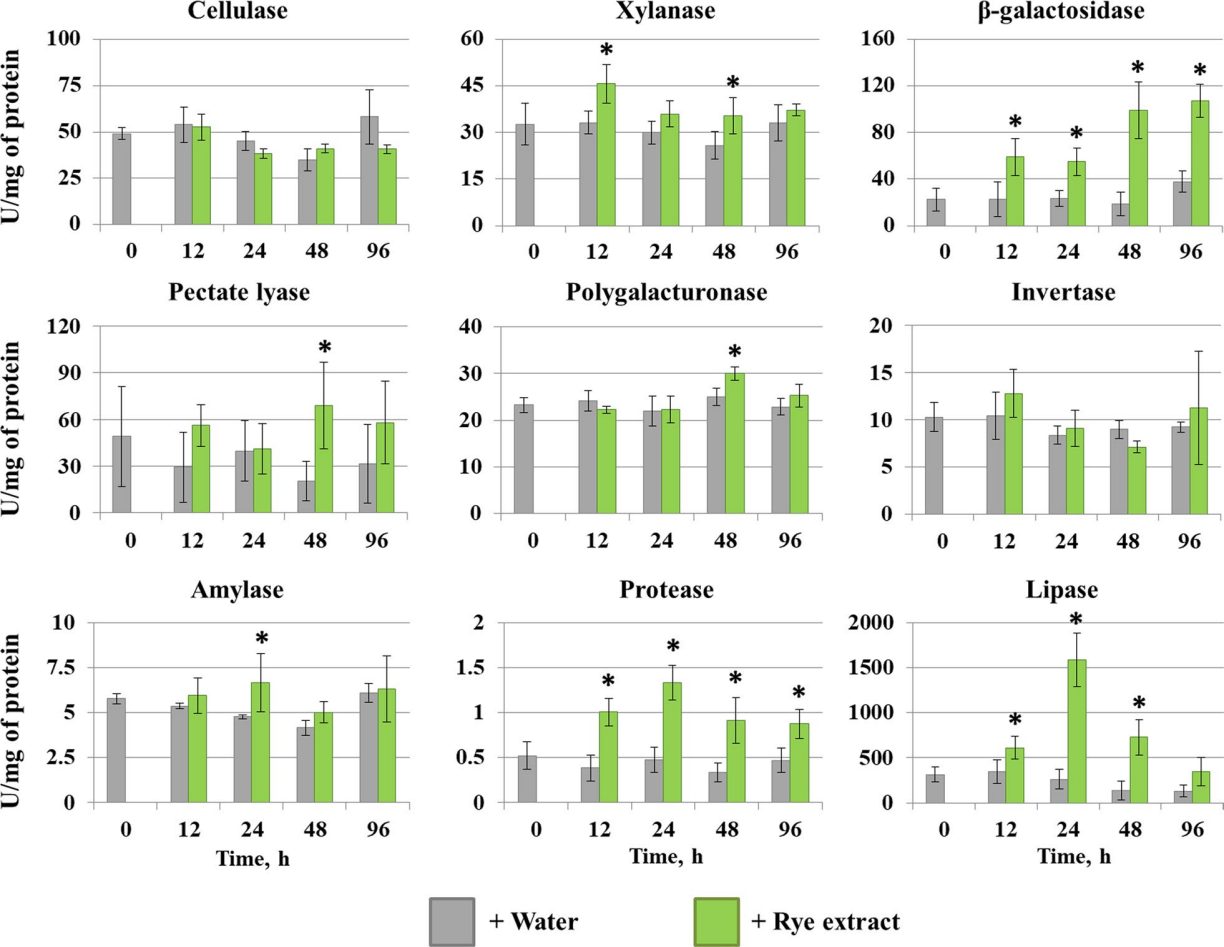
The effect of the extract of rye leaves on the extracellular cellulase, xylanase, β-galactosidase, pectate lyase, polygalacturonase, invertase, amylase, protease, and lipase activities of the *Microdochium nivale* F_00608. Fungal cultures (grown for 10 days in minimal medium) were supplemented with rye extract (green columns) or water (gray columns) and then the enzymatic activities were measured. The presented values are averages ± SD of five biological replicates. The stars show significant differences (Mann–Whitney U test, p-value < 0.05) between samples with and without rye extract

No significant increase in extracellular enzymatic activities related to the degradation of the plant cell wall polysaccharides (cellulase, xylanase, polygalacturonase, pectate lyase) after the addition of rye extract was observed, with the exception of β-galactosidase activity that gradually increased after the supplementation of cultures with the host plant metabolites (Fig. 6). The extracellular amylase and invertase activities were also not induced in the presence of rye extract. In turn, the protease and lipase activities were responsive to the host plant metabolites (Fig. 6). The peak of the induction of these two activities was observed at 24 h after the addition of the host plant extract. Therefore, for the transcriptome profiling, we chose samples collected at 24 h after the addition of rye extract (or water).

The raw RNA-Seq reads generated in this study and used for revealing *M. nivale* genes differentially expressed in response to host plant extract are available at the NCBI BioProject under the accession number PRJNA785089. In total, 10,823 of 11,789 mRNA-encoding *M. nivale* genes were expressed under the experimental conditions. Among them, 1,807 genes were expressed differentially in *M. nivale* depending on the presence of the host plant extract: 872 genes were up- and 935 were downregulated by the host plant metabolites. The functional classification of *M. nivale* genes performed in our study was applied for the description and interpretation of transcriptome profiling data.

No genes encoding cellulases or pectin-degrading enzymes were upregulated (Additional file 3: Table S2), which was in accordance with the results of the enzymatic activity assays (Fig. 6). However, we revealed the upregulation of eight genes encoding another type of cellulose-degrading enzyme—cellulose monooxygenase (Additional file 3: Table S2). Two genes encoding xylanases were upregulated (Additional file 3: Table S2); however, their upregulation was not reflected in the xylanase activity (Fig. 6). Within the supercategory ‘Primary metabolism’, most of the upregulated genes belonged to the categories ‘Amino acid metabolism’, ‘Lipid synthesis/degradation’, and ‘Protein degradation’ (Additional file 3: Table S2). Within the category ‘Amino acid metabolism’, most of the upregulated genes were related to the synthesis of Phe, Tyr, Trp, Ala, Asp, Glu, Arg, and Pro (Additional file 3: Table S2). The upregulation of protein-degradation-related genes as well as genes related to lipid metabolism, including those encoding lipases (Additional file 3: Table S2), was in accordance with the enzymatic activity assays (Fig. 6). Genes related to the transport (supercategory ‘Transport’) of amino acids and lipids were also revealed among the upregulated genes, supporting the plant metabolite-induced alterations in amino acid and lipid metabolism in *M. nivale* (Additional file 3: Table S2).

Within the supercategory ‘Secondary metabolism’, the four upregulated genes encode salicylate hydroxylases (Additional file 3: Table S2)—the enzymes that degrade the defense phytohormone salicylic acid (Qi et al. 2019) and one upregulated gene encodes ent-kaurene oxidase—one of the enzymes for gibberellic acid biosynthesis (Swain and Singh 2005). In addition, seven upregulated genes encoding polyketide synthases were revealed. Among the mycotoxin-related genes, including those that were assembled by us into *M. nivale* mycotoxin-biosynthetic pathways (Fig. 4, Additional file 6: Table S5), only a few genes were upregulated (Additional file 3: Table S2), indicating that the leaf extract hardly induced the production of mycotoxins in *M. nivale*.

Genes related to the transport of sugars and iron (including those encoding ferric-chelate reductases, iron transporter MirB, and genes putatively related to the synthesis of siderophores) as well as vesicle transport were found among the upregulated genes (Additional file 3: Table S2). Within the supercategory of ‘Stress’, genes related to multidrug resistance (MFS transporters), programmed cell death (including the autophagy related proteins), xenobiotic detoxification were upregulated (Additional file 3: Table S2). In the latter category, genes encoding N-hydroxyarylamine O-acetyltransferases, dienelactone hydrolase family protein, and catechol 1,2-dioxygenase that degrade plant defense compounds and are required for virulence (Gardiner et al. 2012; Hammerbacher et al. 2013; Karagianni et al. 2015; Wadke et al. 2016; Soal et al. 2022) were upregulated. The expression of 108 genes encoding transcription factors was responsive to the presence of rye extract: 81 and 27 of them were up- and downregulated, respectively.

## DISCUSSION

Our study presents the first genome-scale analysis of the pink snow mold causal agent, *M. nivale*. In northern countries (with prolonged snowy winters), this disease often reaches the epiphytotic level and, in some years, during wintering and early spring growth, may lead to almost total loss of winter cereals (the most important staple food crops) and also cause severe damage to forage and turf grasses (Dubas et al. 2011; Ponomareva et al. 2021; Temirbekova et al. 2022). The demand for *M. nivale*-related studies is constantly growing, not only because this disease has progressed in the northern countries during the past decade and its management is a serious challenge, but also because *M. nivale* adapts well to less snowy areas, expanding its geography and causing other types of diseases throughout the vegetative season (Tronsmo et al. 2001; Gagkaeva et al. 2017; 2019; Ponomareva et al. 2021). Nevertheless, the *M. nivale* genome was unavailable and no genetic determinants of the virulence of this fungus had been revealed or predicted.

By combining Oxford Nanopore and Illumina technologies, we assembled the 37 Mb genome of a highly virulent *M. nivale* strain F_00608 isolated from diseased winter rye (Gorshkov et al. 2020). Based on the mapping of RNA-Seq reads, 11,789 protein-encoding genes were identified and annotated using different databases. The size and the number of genes in the *M. nivale* genome were in accordance with the corresponding values for the genomes of other representatives of Ascomycota (Schardl et al. 2013; Baker et al. 2015; King et al. 2015; David et al. 2016) and 97.6% of single-copy orthologs of class Sordariomycetes (BUSCO database) were found in the assembled *M. nivale* genome.

To better characterize the *M. nivale* genome structure and to create a convenient template for the description and interpretation of transcriptomic data on this species, the identified genes were split into hierarchical three-level functional categories. The table with a functional gene classification is available (Additional file 3: Table S2) and can be widely used in further *M. nivale* transcriptome studies.

To predict the virulence factors of *M. nivale*, we used three approaches. First, we searched (using PHI-base) for *M. nivale* gene products similar to those that were shown to contribute to the virulence of other cereal phytopathogens from Ascomycota. In *M. nivale*, we revealed 361 such gene products, most of which belonged to the supercategories ‘Primary metabolism’, ‘Cell transport’, and ‘Signaling’. Second, we predicted the secretome of *M. nivale* since many known fungal virulence factors are secreted proteins. Four hundred *M. nivale* gene products were attributed to the secreted proteins, among which many enzymes that digest plant metabolites, including plant cell wall polymers, were found.

Third, we searched for the pathways related to mycotoxin synthesis in *M. nivale*. Although *M. nivale* is considered a species that does not produce mycotoxins (Chełkowski et al. 1991; Nakajima and Naito 1995; Tronsmo et al. 2001; Gagkaeva et al. 2020), we noticed that many genes were annotated as mycotoxin-related ones (Additional file 3: Table S2, sheet ‘Secondary metabolites’). We found that *M. nivale* had the pronounced genetic potential to produce fumonisin, ochratoxin B, aflatoxin, and gliotoxin since all or almost all the genes required for the synthesis of these mycotoxins were identified in the *M. nivale* genome. The pathways related to the synthesis of trichothecene toxins, patulin, zearalenone, and ochratoxin A were incomplete. We also revealed a gene encoding the trichothecene efflux pump TRI12 (MN608_02397) (Alexander et al. 1999; Perlin et al. 2014). This suggests that *M. nivale* can presumably defend itself against trichothecene toxin-producing *Fusarium* species that share an ecological niche (e.g. spikes with head blight symptoms) with *M. nivale* (Nielsen et al. 2013; Karlsson et al. 2021). Herewith, single *M. nivale* toxin-related enzymes (encoded in the *M. nivale* genome) may deactivate the toxin while the efflux pump TRI12 provides its ejection from the cell.

The list of the *M. nivale* mycotoxin-related gene products contains 140 proteins, including enzymes, transcription factors, and transporters. The inconsistency between the presence of mycotoxin-biosynthetic pathways (this study) and undetectable levels of mycotoxins in *M. nivale* cultures (Tronsmo et al. 2001; Chełkowski et al. 1991; Nakajima and Naito 1995; Gagkaeva et al. 2020) can be explained by the following. The synthesis of mycotoxins might not be induced under conditions that were applied for searching for them, and therefore, these compounds were not produced despite the presence of all necessary genes. This is in accordance with the fact that in our study, mycotoxin-related genes were not upregulated by the host plant extract. The factors that induce the expression of these genes and the production of the corresponding compounds remain to be determined. Altogether, three approaches for the identification of the potential virulence factors of *M. nivale* yielded 886 proteins, which deserve special attention in terms of assessing their roles in *M. nivale* virulence (Additional files 4, 5, 6:Table S3-S5).

Before our study, the only representative of the *Microdochium* genus with a sequenced genome was *M. bolleyi* (David et al. 2016). Interestingly, although *M. bolleyi* is a non-phytopathogenic species, we revealed that almost all genes encoding potential virulence factors are shared between *M. nivale* and *M. bolleyi*. The exceptions were genes encoding hydroxyversicolorone monooxygenases and cytochrome P450 monooxygenases (related to the biosynthesis of aflatoxin and aspirochlorine, respectively) that were present in *M. nivale* but not in *M. bolleyi*. Such similarity in a set of genes for potential virulence factors in closely related phytopathogenic and non-phytopathogenic species can be explained by the following. First, it cannot be excluded that it is indeed aflatoxin and aspirochlorine that are crucial for the pathogenicity of *M. nivale*, while *M. bolleyi*, lacking some of the aflatoxin-and aspirochlorine-related genes, is unable to cause disease. Second, it is possible that the deferential pathogenicity of *M. nivale* and *M. bolleyi* is determined not by the set of virulence factors but by the regulatory networks coordinating their production. Third, although *M. bolleyi* has been described as a non-phytopathogenic root endophyte (Rothen et al. 2018), it cannot be ruled out that, under specific conditions, it is also able to cause disease and thus manifest phytopathogenicity.

The *M. nivale* gene set also displayed high similarity with those of some other phytopathogenic fungi, both psychrophilic (*Sclerotinia borealis*) and mesophilic (*Fusarium graminearum*) parasitizing on cereals. In particular, almost all genes for potential virulence factors were shared among the three species, excluding 28 P450 oxygenases putatively related to the biosynthesis of aspirochlorine that were unique to *M. nivale*. The comparison of the three genomes did not reveal gene products whose functions could explain (at least speculatively) the psychrotolerance of *M. nivale*. Thus, it is likely that the cold tolerance of *M. nivale* results from either gene regulation or nuances in the structure of different enzymes/proteins that contribute to their functioning under low temperatures, or both.

The assembled and characterized *M. nivale* genome was further used in our study as a reference for comparative transcriptome profiling. Host plant extracts are well-known to contain metabolites that signal to pathogens that it is reasonable to spend resources for the realization of their phytopathogenic potential, leading to the manifestation of virulence-related traits (Brencic and Winans 2005; Mattinen et al. 2007; Tarasova et al. 2013). Therefore, we assessed the effect of the host plant metabolites (rye extract) on the transcriptome profile of *M. nivale*. The used in vitro model allowed us to analyze possible host metabolite-induced alterations in not only *M. nivale* gene expression levels but also fungal extracellular enzymatic activities (major virulence factors), enabling the comparison of the results of the transcriptomic and biochemical studies.

The supplementation of the *M. nivale* cultures with rye extract led to the upregulation of genes related to lipid metabolism, including genes encoding lipases and lipid transporters, as well as strongly induced extracellular lipase activity. Alterations in lipid metabolism resulting in the accumulation of triacylglycerols and unsaturated lipids in hyphae are considered to determine *M. nivale* psychrotolerance (Istokovics et al. 1998; Okuyama et al. 1998). In turn, the host plant likely promotes these alterations. In the course of *M. nivale* infection, both lipase-encoding genes of the plant and lipase activity in infected plants are induced (Tsers et al. 2021). In this case, the increase in the breakdown of lipids by plant enzymatic machinery was proposed to be a result of plant susceptible response—host reaction driven by the pathogen manipulation in order to “improve” its ecological niche and, in particular, to obtain necessary nutrients by exploiting host plant enzymes (Gorshkov and Tsers 2022). In our present study, we show that, in addition to the exploitation of the host plant lipases, *M. nivale* may use its own extracellular lipases, whose activity is induced by the water-extractable host plant metabolites, to promote the release of lipid precursors from plant tissues. This further supports the hypothesis that lipids are one of the crucial “targets” for *M. nivale* within the host plants, and alterations in lipid metabolism play an important role in *M. nivale*-plant interactions.

Similar to the “lipid story”, both protease-encoding genes and extracellular protease activity were induced in *M. nivale* by the host plant extract (this study), while protease-encoding genes and protease activity were induced in rye plants following *M. nivale* infection (Tsers et al. 2021). Additionally, genes related to the synthesis and transport of amino acids (Phe, Tyr, Trp, Ala, Asp, Glu, Arg, and Pro) were upregulated after supplementation of fungal cultures with rye extract, pointing to alterations in the amino acid metabolism. However, the specific role of the consumption of the host plant protein-degradation product in *M. nivale* fitness is less evident compared to the consumption of lipids.

Except for β-galactosidase activity, none of the assayed plant cell wall polysaccharide-degrading extracellular enzymatic activities (cellulase, xylanase, polygalacturonase, pectate lyase) were induced in *M. nivale* cultures after the addition of rye extract, which was consistent with the expression pattern of corresponding genes. Since these enzymes seem important for causing the disease, it is likely that their activities (and the expression of corresponding genes) are induced by water-insoluble components of plant tissues. In turn, genes encoding one type of the plant cell wall degrading enzymes—cellulose monooxygenases—were upregulated in the presence of rye extract. Cellulose monooxygenases belong to the recently discovered lytic polysaccharide monooxygenases (LPMOs) that are able to oxidize polysaccharide chains packed into crystalline lattices (that are hardly accessible to most enzymes), creating chain ends tractable by canonical glycosyl hydrolases for further depolymerization (Vandhana et al. 2022). It is highly expected that LPMOs can contribute to phytopathogens in the degradation of the host plant polysaccharides, including cellulose. However, in terms of plant-pathogen interaction, to date, these enzymes, which are active toward not only crystalline but also amorphous polysaccharides as well as toward oligosaccharides, have been described only as tools to ‘mask’ chito-oligosaccharides released from the fungal cell wall (Li et al. 2020). In this case, LPMO-mediated modification of chito-oligosaccharides prevents their recognition by plant receptors, allowing the pathogen to evade the host immune system. The role of these recently discovered enzymes in the virulence of phytopathogens seems important and multifunctional, especially given the representativeness of LPMO-encoding genes in fungal genomes (Vandhana et al. 2022). In *M. nivale*, we revealed 23 LPMO-encoding genes (including 19 genes encoding cellulose monooxygenases); 8 of these genes were upregulated in the presence of rye extract.

*M. nivale* is presumably able to interfere with the host plant's hormonal systems. In the *M. nivale* genome, we revealed many genes encoding the enzymes that cleave salicylic acid and auxin, as well as gibberellin-biosynthetic enzymes, and some of these genes were upregulated following the addition of rye extract to the culture medium.

Rye extract induced the expression of stress-related genes, including those encoding MFS transporters and xenobiotic-detoxifying enzymes. MFS transporters, in addition to sugar influx (Pereira et al. 2013; Ramón-Carbonell and Sánchez-Torres 2017; Vela-Corcía et al. 2019), may also efflux toxic compounds and thus promote fungicide resistance (Costa et al. 2014; Popiel et al. 2017) and interspecific competition (Malmierca et al. 2013). These transporters have also been shown to be responsible for the efflux of plant antimicrobial compounds (isothiocyanates) from *Botrytis cinerea* (Vela-Corcía et al. 2019) and for promoting the virulence of many phytopathogenic fungi (Hayashi et al. 2002; Choquer et al. 2007; Roohparvar et al. 2007; Omrane et al. 2017; Popiel et al. 2017; Vela-Corcía et al. 2019; Diao et al. 2021).

The detoxication of plant antimicrobial compounds may be provided by fungal enzymes, including N-hydroxyarylamine O-acetyltransferase (NAT) (Karagianni et al. 2015), dienelactone hydrolase (Gardiner et al. 2012), catechol 1,2-dioxygenase (Hammerbacher et al. 2013; Wadke et al. 2016). *M. nivale* genes encoding these three enzymes were upregulated in the presence of rye extract (this study). NAT genes required for *Fusarium* species to detoxicate benzoxazinoids—phytoalexins found in many *Poaceae* species, including rye (Karagianni et al. 2015; Hazrati et al. 2022), were strongly upregulated (log_2_FC 5.8 and 3.4) in *M. nivale* after the addition of rye extract (this study). In our previous study, we showed that plant genes encoding the enzymes that catalyze the formation of the benzoxazinoid phytoalexin DIMBOA were upregulated in rye following *M. nivale* infection (Tsers et al. 2021). In turn, our present study shows that the rye extract induces the expression of *M. nivale* genes whose products may provide detoxication of this antimicrobial compound in fungal hyphae, protecting the pathogen from the host plant's defense response.

In addition to the rye extract-upregulated *M. nivale* genes encoding transporters of sugars and xenobiotics, including mycotoxins, genes related to the transport of iron were also upregulated, as were vesicular transport-related genes. Iron is a cofactor of many enzymes, including virulence factors, and is required for many other virulence-related processes (Renshaw et al. 2002; Ding et al. 2014; Saikia et al. 2014). Phytopathogenic fungi have evolved at least two iron acquisition systems: (1) mediated by iron scavengers—siderophores; and (2) mediated by cell wall localized ferric-chelate reductases that reduce insoluble ferric iron to soluble ferrous iron, enabling its uptake by transporters (Greenshields et al. 2007). In our study, genes encoding ferric-chelate reductases, iron transporter MirB, and genes putatively related to the synthesis of siderophores were upregulated after the addition of rye extract, indicating that the presence of host plant metabolites promoted iron uptake by fungal cells. Many virulence factors are transported from fungal cells via extracellular vesicles (Silva et al. 2014; Bleackley et al. 2019; Cairns et al. 2021; Garcia-Ceron et al. 2021), including those that lack signal peptides (Kijpornyongpan et al. 2022). This means that extracellular vesicular transport is important for plant-pathogen interaction. Our study shows that the host plant metabolites induce the expression of vesicular transport-related genes in *M. nivale*, presumably facilitating the realization of its virulence program.

Our study reveals many important aspects of *M. nivale* virulence in terms of both genetic potential and its realization under host plant-mimicking conditions. In particular, whole-genome sequencing and annotation showed a high genetic potential of *M. nivale* to produce human-dangerous mycotoxins that were previously unrevealed in this species by biochemical approaches (Chełkowski et al. 1991; Nakajima and Naito 1995; Tronsmo et al. 2001; Gagkaeva et al. 2020). In addition to mycotoxin-related genes, our study predicted a number of genes whose products seem to be the most apparent virulence factors of *M. nivale* that need to be further analyzed by various approaches. Simultaneously, it is evident that the analysis using a single test system at a single time point cannot allow an understanding of the global picture of the *M. nivale*-plant interaction. Further accumulation of data on alterations in *M. nivale* transcriptome profiles and other physiological parameters under the influence of different fractions of host plant metabolites, including the insoluble ones, and under *in planta* conditions at different stages of infection and different plant compartments will improve the understanding of the major set of *M. nivale* virulence determinants and mechanisms of the regulation of their production by both host plant metabolites and fungal regulatory networks. Our study forms a valuable foundation for implementing these investigations by presenting not only the reference *M. nivale* genome but also a detailed functional gene classification that will allow the researchers to significantly facilitate, improve, detail, and conceptualize the information on the differential gene expression in *M. nivale*.

In addition to transcriptomic studies directed toward the genome-wide analysis of physiological responses of *M. nivale*, the reference *M. nivale* genome will serve as a stimulus for undertaking pangenome studies to better understand intraspecific diversity, which is strongly pronounced in *M. nivale* species in terms of not only genetic but also physiological aspects, including virulence and host specificity (Mahuku et al. 1998; Abdelhalim et al. 2020; Gorshkov et al. 2020). This diversity is reflected, in particular, in a large number of HET genes (133 genes identified in the genome assembly) whose products control heterokaryon incompatibility during the fusion of hypha of genetically diverse fungal strains (Bégueret et al. 1994). *M. nivale* pangenome investigations have great importance since they can aid in the differentiation of highly and low-aggressive strains or strains with different host specificity. In addition, the comparison of the genomes of different *M. nivale* strains from different territories will contribute to the understanding of how this phytopathogen adapts to a warmer climate spreading beyond its snowy area, causing different types of plant diseases.

## CONCLUSIONS

The first genome of the pink snow mold causal agent, *M. nivale*, has been assembled and annotated. The detailed functional classification of *M. nivale* genes (available for general use (Additional file 3: Table S2)) has been performed, contributing to further deep analyses of *M. nivale* responses at a whole genome level and pangenome studies. *M. nivale* gene products that best meet the criteria for virulence factors have been identified. The genetic potential to synthesize human-dangerous mycotoxins has been revealed for *M. nivale*. The arguments in favor of a crucial role of host plant lipid destruction and fungal lipid metabolism modulation in plant-*M. nivale* interactions have been obtained using transcriptome profiling and enzymatic assays. The obtained results are valuable for further genetic and phytopathological studies on *M. nivale* as well as for the targeted resistance breeding of cereal crops.

## Supplementary Information

**Additional file 1**. **Figure S1**: Trichothecene, patulin, and zearalenone mycotoxin biosynthetic pathways showing pathway-related gene products predicted in the Microdochium nivale reference proteome. Rectangles contain names of the reference proteins involved in the synthesis of corresponding mycotoxins, including those for which orthologs were revealed (green) or were not revealed (grey) in M. nivale proteome. The IDs of genes for the revealed orthologs are given near the corresponding rectangles.

**Additional file 2**. **Table S1**: The summary of the results of the sequencing of the Microdochium nivale genomic DNA on the platforms of Nanopore (long reads, sheet “Nanopore”) and Illumina (short reads, sheet “Illumina”), as well as M. nivale RNA-Seq analysis (sheet “RNA-Seq”).

**Additional file 3**. **Table S2**: The functional classification of Microdochium nivale protein-encoding genes. The classification is subdivided into the supercategories (designated in the corresponding sheets), which are split into (sub)categories given within each sheet. FCW—fungal cell wall.

**Additional file 4**: **Table S3**: The list of Microdochium nivale genes encoding proteins similar (the BLAST hits of alignment query coverage ≥70% and sequence similarity ≥60%) to the virulence factors of phytopathogenic Ascomycota selected from the PHI-base.

**Additional file 5**. **Table S4**: The list of Microdochium nivale genes encoding secreted proteins predicted using both SignalP5.0 and Secretool.

**Additional file 6**. Table S5: Microdochium nivale genes encoding proteins orthologous to the reference proteins involved in mycotoxin biosynthesis in various Ascomycota species.

**Additional file 7**. **Table S6**: The results of the comparison of the reference proteomes of Microdochium nivale and Microdochium bolleyi. Shared orthologues and proteins unique to each species are listed in the corresponding sheets.

**Additional file 8**. **Table S7**: The results of the comparison of the reference proteomes of Microdochium nivale, Sclerotinia borealis, and Fusarium graminearum. Shared orthologues and proteins unique to M. nivale are listed in the corresponding sheets.

## Data Availability

The DNA and RNA sequencing reads generated in this study are available at NCBI BioProjects PRJNA808256 (the genomic reads used for assembly and the RNA-Seq reads used for revealing the mRNA-encoding genes) and PRJNA785089 (the RNA-Seq reads used for differential gene expression analysis). The genome assembly is available under the WGS accession JANTFD000000000 (or alternatively at https://doi.org/10.17605/OSF.IO/2TKUF). Genome functional classification is available as the Additional file 3: Table S2 (this study).
