# Supplementary figures and images for: First genome-scale insights into the virulence of the snow mold causal fungus Microdochium nivale
Source: IMA Fungus. 2023 Jan 10;14:2. doi: 10.1186/s43008-022-00107-0 (PMC9830731; doi:10.1186/s43008-022-00107-0)

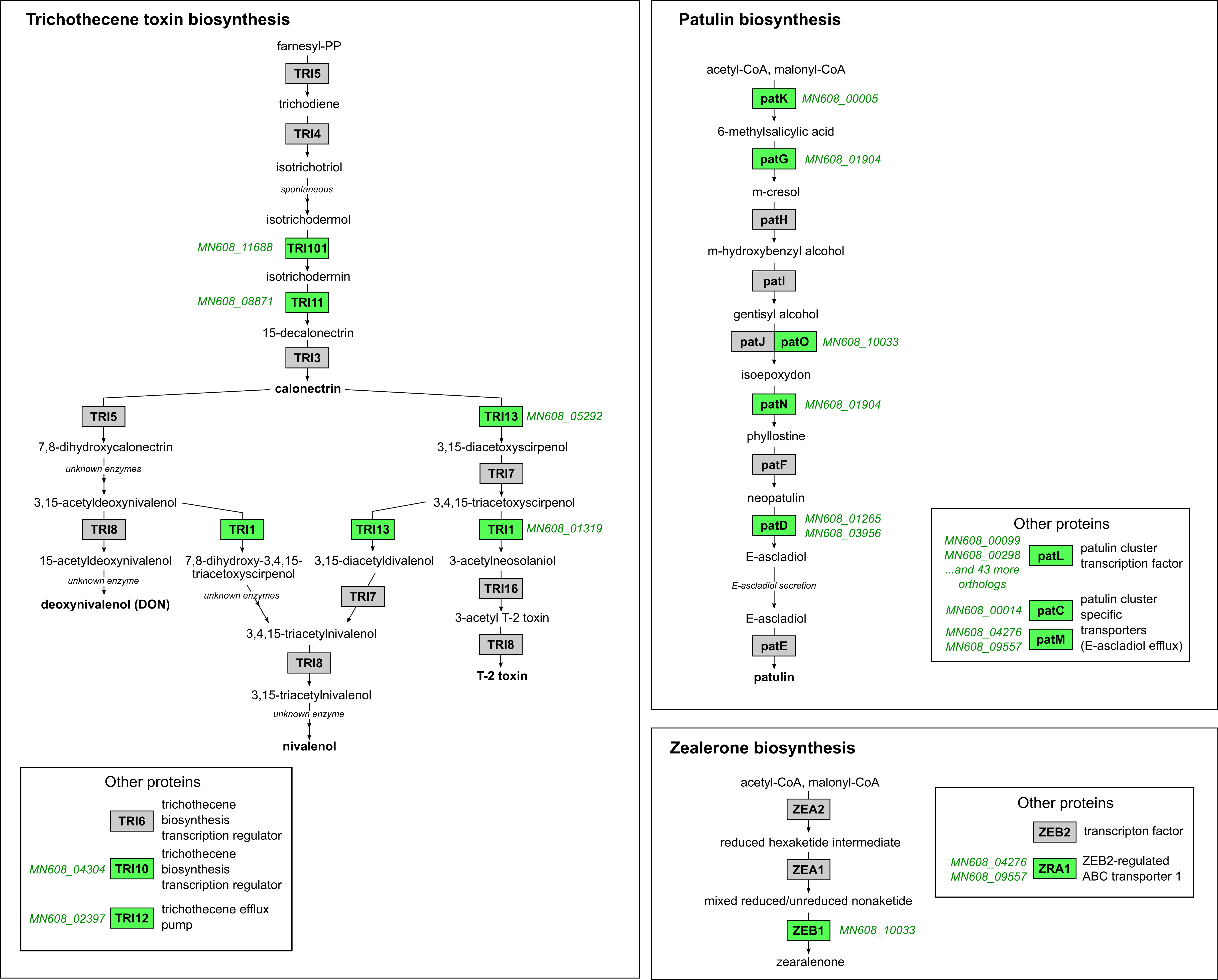

Supplement: Supplementary file 1 — Additional file 1. Figure S1: Trichothecene, patulin, and zearalenone mycotoxin biosynthetic pathways showing pathway-related gene products predicted in the Microdochium nivale reference proteome. Rectangles contain names of the reference proteins involved in the synthesis of corresponding mycotoxins, including those for which orthologs were revealed (green) or were not revealed (grey) in M. nivale proteome. The IDs of genes for the revealed orthologs are given near the corresponding rectangles. [file 43008_2022_107_MOESM1_ESM.png]
